# Supplementary material for: Multistep transition of diamond to warm dense matter state revealed by femtosecond X-ray diffraction
Source: Sci Rep. 2018 Mar 27;8:5284. doi: 10.1038/s41598-018-23632-8 (PMC5869726; doi:10.1038/s41598-018-23632-8)
Supplement: Supplementary file 1 — Supplementary Information [file 41598_2018_23632_MOESM1_ESM.docx]

## **Supplementary Information**

For “Multistep transition of diamond to warm dense matter state revealed by femtosecond X-ray diffraction”

N. Medvedev^[[1]](#footnote-1),1,2^ and B. Ziaja^[[2]](#footnote-2)^,^3,4^

(1) Institute of Physics, Czech Academy of Sciences, Na Slovance 2, Prague 8, 18221, Czech Republic

(2) Institute of Plasma Physics, Czech Academy of Sciences, Za Slovankou 3, Prague 8, 18200, Czech Republic

(3) Center for Free-Electron Laser Science, DESY, Hamburg, 22607, Germany

(4) Institute of Nuclear Physics, Polish Academy of Sciences, Krakow, 31-342, Poland

1. **Duration of existence of transient graphite-like state**

We performed a study of the transient graphite-like phase formation following the irradiation of diamond with a high-intensity X-ray pulse. From our earlier investigations, we know that the graphitization in irradiated diamond occurs if the average dose absorbed per atom exceeds ~0.7 eV/atom [1,2]. The graphitization timescale is then ~150-200 fs [3] (at the pump pulse duration of 50 fs). Pure thermodynamic consideration implies that if we deliver to the electronic system in diamond such an amount of energy and let the system evolve, the graphitization has to occur.

The situation in the analyzed experiment [4] is different, as the pump pulse is so intense that, after crossing the graphitization threshold, much energy is still quickly absorbed by the system, resulting in the final dose of 18-25 eV per atom. Thus, after crossing the graphitization threshold, the system cannot evolve freely: its further evolution is perturbed by the processes accompanying the on-going energy absorption. Then the question is, can the transient graphite-like phase still be observed?

One can expect that the transient graphite-like sp^2^-carbon phase would manifest stronger if the interplay of energy absorption processes and the phase transition can be diminished. This can be achieved if the pump pulse duration would be longer at the same absorbed pulse fluence.

Supplementary Figure 1. Dose absorbed per atom as a function of time in diamond irradiated with FEL pulses of 5 fs and 100 fs FWHM duration, both yielding a maximal absorbed dose of 18.5 eV/atom.

Indeed, Supplementary Figure 1 shows the absorbed energy per atom in diamond under irradiation with an FEL pulse of 5 fs or 100 fs FWHM. The damage thresholds for graphitization (0.7 eV/atom) and for graphite damage (additional ~3.3 eV/atom) are indicated with dashed lines. We can see that for 100 fs long pulse, the absorbed dose stays in the vicinity of the graphitization threshold for a certain time (~ few tens fs), during which a formation of graphite-like state occurs. If the energy absorption continues, this state evolves further towards a disordered state. In comparison, for 5 fs pulse, the system remains within the ‘graphite energy window’ only for a few femtoseconds.

Accordingly, we performed a simulation for a longer FEL pulse of 100 fs FWHM. This allows to reduce the influence of the processes accompanying the on-going energy absorption (such as electronic excitation, secondary electron cascading etc.) on the on-going phase transition. For the FEL parameters corresponding to the experimental conditions [4] (5 fs long pump pulse), these processes strongly influence sample evolution, due to their high rates. For 100 fs long pulse, their rates are lower, and the graphite-like phase can be indeed observed for longer times. Supplementary Figure 2 shows the diffraction patterns of diamond under irradiation with 100 fs long FEL pulse. The patterns confirm that a graphite-like structure indeed forms during some 30 fs, before a complete disorder of the atomic system occurs.

**

Supplementary Figure 2. Diffraction patterns of diamond irradiated with an FEL pulse of 6.1 keV photon energy and 100 fs FWHM duration, yielding a maximal absorbed dose of 18.5 eV/atom: (a) at different instants of time; (b) a zoom onto the graphite-like peak at the time instant of -10 fs.

For comparison, results of the simulation of irradiated diamond at the same deposited dose with the pulse duration of 5 fs (Fig. 2 in the main text) are shown zooming on the transient graphite-like peak, Supplementary Figure 3. It confirms the transient emergence of the graphite-like peak in case of short FEL pulse. This peak is, albeit being small, clearly above the noise level. As expected, the duration of the graphite-like phase is much shorter than in the case of 100 fs FEL pulse.

The current experimental data follow only the evolution of peaks (111) and (220). We argue in the main text that the ratio of these peaks can be considered indicative of the transient graphite-like phase, however, additional dedicated experiments is necessary to verify the occurrence of the graphite-like peak. Therefore, current predictions can be considered a hypothesis to be verified.

Supplementary Figure 3. Diffraction patterns of diamond irradiated with an FEL pulse of 6.1 keV photon energy and 5 fs FWHM duration, yielding the maximal absorbed dose of 18.5 eV/atom: a zoom onto the graphite-like peak at the time instant of 23 fs (from Fig. 2 in the main text).

**References**

[1] N. Medvedev, H. O. Jeschke, and B. Ziaja, New Journal of Physics **15**, 15016 (2013).

[2] J. Gaudin, N. Medvedev, J. Chalupský, T. Burian, S. Dastjani-Farahani, V. Hájková, M. Harmand, H. O. Jeschke, L. Juha, M. Jurek, D. Klinger, J. Krzywinski, R. A. Loch, S. Moeller, M. Nagasono, C. Ozkan, K. Saksl, H. Sinn, R. Sobierajski, P. Sovák, S. Toleikis, K. Tiedtke, M. Toufarová, T. Tschentscher, V. Vorlíček, L. Vyšín, H. Wabnitz, B. Ziaja, and E. Al., Physical Review B **88**, 060101(R) (2013).

[3] F. Tavella, H. Höppner, V. Tkachenko, N. Medvedev, F. Capotondi, T. Golz, Y. Kai, M. Manfredda, E. Pedersoli, M. J. Prandolini, N. Stojanovic, T. Tanikawa, U. Teubner, S. Toleikis, and B. Ziaja, High Energy Density Physics **24**, 22 (2017).

[4] I. Inoue, Y. Inubushi, T. Sato, K. Tono, T. Katayama, T. Kameshima, K. Ogawa, T. Togashi, S. Owada, Y. Amemiya, T. Tanaka, T. Hara, and M. Yabashi, Proceedings of the National Academy of Sciences of the United States of America **113**, 1492 (2016).

1. Corresponding author: nikita.medvedev@fzu.cz [↑](#footnote-ref-1)
2. Corresponding author: ziaja@mail.desy.de [↑](#footnote-ref-2)
